# The Effect of Vitamin D Supplementation on Functional Outcomes in Patients Undergoing Rehabilitation After an Ischemic Stroke: A Prospective, Single-Blind, Randomized, Placebo-Controlled Study

**DOI:** 10.3390/jcm14061848

**Published:** 2025-03-09

**Authors:** Wojciech Borowicz, Lucyna Ptaszkowska, Rafał Małecki, Małgorzata Paprocka-Borowicz

**Affiliations:** 1 Department of Pediatric Infectious Diseases, Wroclaw Medical University, 50-368 Wroclaw, Poland; wojciech.borowicz@umw.edu.pl; 2 Institute of Health Sciences, University of Opole, 45-060 Opole, Poland; lucyna.ptaszkowska@uni.opole.pl; 3 Department of Non-Procedural Clinical Science, Faculty of Medicine, Wroclaw University of Science and Technology, 51-377 Wroclaw, Poland; rafal.malecki@pwr.edu.pl; 4 Department of Internal Medicine with Angiology Subdivision, Regional Specialist Hospital in Wroclaw, 51-124 Wroclaw, Poland; 5 Division of Clinical Physiotherapy and Rehabilitation, University Centre of Physiotherapy and Rehabilitation, Faculty of Physiotherapy, Wroclaw Medical University, 50-368 Wroclaw, Poland; 6 Department of Neurological Rehabilitation, Regional Specialist Hospital in Wroclaw, 51-128 Wroclaw, Poland

**Keywords:** ischemic stroke, vitamin D3 supplementation, rehabilitation, Barthel index, modified Rankin scale, neuroplasticity, randomized controlled trial

## Abstract

**Background/Objectives**: A vitamin D deficiency is prevalent in post-stroke patients and may impair neurological recovery. While observational studies highlight the neuroprotective role of vitamin D, there is limited evidence from interventional studies evaluating its impact on functional recovery during stroke rehabilitation. This study aimed to assess whether daily vitamin D3 supplementation enhances functional recovery. **Methods**: This prospective, randomized, placebo-controlled, single-blind study included 159 patients (mean age: 62.5 ± 8.4 years) with a first ischemic stroke that were admitted for early rehabilitation. The participants were randomly allocated to receive 2000 IU of vitamin D3 daily (*n* = 79) or a placebo (*n* = 80) for six weeks. The functional outcomes were measured using the Barthel index (BI) and modified Rankin scale (mRS) at baseline and after 42 days. The serum 25-hydroxyvitamin D [25(OH)D] and insulin-like growth factor 1 (IGF-1) levels were analyzed. **Results**: Vitamin D3 supplementation significantly increased the serum 25(OH)D levels (*p* < 0.001). Supplementation was associated with improved BI scores (β = 0.07, *p* = 0.006). A higher BMI (β = −0.06, *p* = 0.033), higher NIHSS scores (β = −0.18, *p* = 0.036), hypertension, and statin use negatively impacted functional recovery. Anticoagulant use was correlated with higher mRS scores, indicating greater disability (*p* = 0.04). **Conclusions**: Vitamin D3 supplementation positively influences the functional outcomes during post-stroke rehabilitation, supporting its potential role in enhancing neuroplasticity and recovery. Larger multi-center trials are needed to confirm these findings and optimize vitamin D supplementation strategies.

## 1. Introduction

Every year, millions of people worldwide experience a stroke, regardless of race, origin, or socioeconomic status [1]. Cerebrovascular diseases, including strokes, rank as the second leading cause of death globally [2]. The full recovery of function and perception occurs in only 10% of patients. Approximately 15% of stroke patients die in the early phase, while 25% experience a recurrent stroke. The risk of recurrence is highest within the first and second years following the initial event. Individuals who have experienced a stroke are at a significantly greater risk of a second stroke compared to their peers of the same age and sex who have not had a stroke [3]. Stroke prevention strategies focus primarily on behavioral and lifestyle factors [4]. Hence, managing risk factors plays a crucial role in medical care. Strokes are the leading cause of disability in individuals over the age of 45 [5].

Stroke timing critically influences the potential for neuroplasticity and functional recovery, particularly during the early phase of rehabilitation. Indeed, recent findings underscore the importance of initiating therapy promptly to leverage optimal brain reorganization [6]. It is generally accepted that cortical reorganization peaks 7–14 days post-stroke and persists for approximately one month [7]. Early rehabilitation reduces disability at the end of the rehabilitation period, lowering future healthcare costs. Global concerns about vitamin D deficiencies are increasing, affecting nearly half of the population worldwide [8,9].

A vitamin D deficiency is now recognized as a public health issue. Recent population-based studies indicate that low vitamin D levels predict future strokes. Poland, characterized by limited sunlight exposure, is among the countries where vitamin D deficiencies are prevalent [10,11]. Low serum 25-hydroxyvitamin D (25[OH]D) levels are linked to cardiovascular, musculoskeletal, infectious, autoimmune, and malignant diseases [12]. Vitamin D has been shown to have neuroprotective, neuromuscular, and osteoprotective properties, potentially reducing cognitive and functional impairments in post-stroke patients [13]. The current literature suggests that vitamin D supplementation and neuroprotective diets can enhance the efficacy of stroke rehabilitation and recovery.

Vitamin D is believed to support neurological function and recovery through several interconnected pathways. First, vitamin D receptors (VDRs) are expressed in various regions of the central nervous system, including the cortex and hippocampus, suggesting that vitamin D can exert both genomic and non-genomic effects on neuronal survival and plasticity [14,15]. In the setting of an ischemic stroke, vitamin D may mitigate neuroinflammation by downregulating pro-inflammatory mediators such as interleukin 6 (IL-6) and tumor necrosis factor alpha (TNF-α), potentially limiting secondary neuronal damage [16,17]. Furthermore, vitamin D has been shown to influence the expression of neurotrophic factors—such as nerve growth factor (NGF) and brain-derived neurotrophic factor (BDNF)—which are crucial for neuronal repair and synaptic plasticity [18]. Additional research also points to an improvement in endothelial function and microcirculatory blood flow under optimal vitamin D levels, contributing to reduced ischemic injury in the affected brain tissue [19]. Collectively, these mechanisms support the hypothesis that an adequate vitamin D status may facilitate improved functional recovery and neurorehabilitation outcomes in post-stroke patients.

However, interventional studies remain scarce, highlighting the urgent need for randomized controlled trials (RCTs) to evaluate the impact of vitamin D supplementation on stroke outcomes [20]. Therefore, this study aimed to determine whether vitamin D3 supplementation influences the functional recovery in patients undergoing a 6-week neurological rehabilitation program during the regenerative–compensatory phase.

## 2. Materials and Methods

### 2.1. Ethics and Consent

This study received approval from the Bioethics Committee of the Wroclaw Medical University (No. KB-813/2023). The trial was registered with the International Standard Randomized Controlled Trial Number (ISRCTN) registry (No. ISRCTN11086312). The study adhered to good clinical practice (GCP) guidelines, evidence-based medicine principles, and the Declaration of Helsinki (DoH) [21]. This was a prospective, randomized, placebo-controlled, single-blind study; therefore, the Consolidated Standards of Reporting Trials (CONSORT) guidelines were implemented [22]. All the participants were informed about the study procedures and provided written informed consent. The patients were allowed to withdraw at any stage due to health issues or personal reasons.

### 2.2. Participants

This study involved 160 participants aged 45–75 who had experienced their first ischemic stroke and were admitted for early post-stroke rehabilitation. The participants were randomly assigned to two groups. The project spanned from March to December 2023. The study population was selected from patients receiving rehabilitation at the Neurological Rehabilitation Department of the Regional Specialist Hospital in Wroclaw, Poland.

### 2.3. Enrollment

The participants were screened and enrolled within two weeks (14 days) post-stroke. The mean elapsed time from stroke onset to the start of rehabilitation was 9.8 ± 2.1 days in the vitamin D3 group versus 10.2 ± 2.3 days in the placebo group (*p* = 0.56), indicating no statistically significant difference in the baseline timing between the two groups. The inclusion criteria included the following: the first ischemic stroke, admission within two weeks post-discharge from neurology or internal medicine departments for early neurological rehabilitation, confirmation of the stroke by CT or MRI, an age > 18 years, the absence of contraindications to participate (physician approval), the absence of concurrent neurological disorders, and signed informed consent. The exclusion criteria comprised the following: infections within the last two weeks, vitamin D or calcium supplementation in the preceding three months, liver or kidney dysfunction, thyroid disorders, global aphasia, or refusal to participate. 

### 2.4. Randomization and Blinding 

A simple randomization sequence was generated using computer software (random.org; accessed on 1 April 2023. The participants were randomly allocated in a 1:1 ratio to either the vitamin D3 supplementation group (2000 IU/day) or the placebo group. Allocation assignments were placed in sequentially numbered, sealed, opaque envelopes, which were opened only after an eligible participant provided informed consent and was enrolled in the study. This was a single-blind trial: the patients (and their caregivers) remained unaware of their group allocation (vitamin D vs. placebo), while the clinical team administering the supplementation was informed to ensure correct dispensing and monitoring. Critically, the principal investigator conducting the outcome assessments (e.g., Barthel index and modified Rankin scale, mRS) was blinded to each participant’s group assignment. This approach minimized assessment bias while ensuring the safety and feasibility of daily supplementation in a clinical rehabilitation setting.

### 2.5. Study Procedures

Eligible participants were randomly assigned to two groups using randomization software (random.org): Group A: daily supplementation with 2000 IU of vitamin D3 at 7:30 AM for six weeks. Group B (control): placebo group without vitamin D3 supplementation. All the participants underwent assessments at hospital admission and after 42 days. A neurological examination, a clinical symptom evaluation (National Institutes of Health Stroke Scale, NIHSS) [23], and functional assessments (Barthel index and mRS) [24] were conducted.

Anthropometric measurements were performed at baseline. Blood samples were collected for routine laboratory tests and biochemical analyses, including the serum 25(OH)D and IGF-1 levels, at baseline and after six weeks of rehabilitation. The blood draws were conducted at 6:30 AM under fasting conditions from the antecubital vein. The vitamin D (25-OH) levels were measured using the ALINITY ci analyzer and ABBOTT reagents. A competitive chemiluminescent microparticle immunoassay (CMIA) quantified the serum 25(OH)D levels. Vitamin D sufficiency was defined as serum 25-(OH)-D > 30 ng/mL, while deficiency was defined as <30 ng/mL. All the tests and questionnaires were administered by the same clinician [25].

### 2.6. Rehabilitation Protocol

The goal of rehabilitation for post-stroke patients is to restore higher-order, cognitive, and motor functions to pre-stroke levels. Both groups underwent identical rehabilitation protocols involving neurophysiological methods (PNF and mirror therapy) five days a week. Each session lasted 60 min. Additionally, all the patients received daily 30 min mirror therapy sessions. The rehabilitation program lasted six weeks. The patients also participated in daily 30 min individual sessions with a clinical psychologist and 45 min occupational therapy sessions. For safety, blood pressure and heart rate were measured before each kinesiotherapy session. In patients with diabetes, the rehabilitation was halted if electrocardiography (ECG) changes suggesting myocardial ischemia occurred during exercise. The rehabilitation was individually tailored to each patient’s current abilities and needs. Early rehabilitation programs for post-stroke patients in our clinical setting generally span 4–6 weeks, aligning with standard practice to maximize neuroplasticity during the subacute phase.

### 2.7. Measurements

The therapeutic progress was assessed using the Barthel index and mRS, which are widely accepted for measuring physical dependency with a high inter-rater reliability. Both scales were applied at admission and after 42 days. A functional disability was defined by an mRS score > 3. This study examined the relationship between the Barthel index and mRS outcomes and the serum vitamin D3 (25-OH) levels at two time points during the regenerative–compensatory phase post-ischemic stroke. The Barthel index, despite its known ceiling effects, is widely recognized and extensively validated for assessing the basic activities of daily living (ADLs). While we understand that some studies use longer durations or additional scales (e.g., FIM, Berg balance scale) to capture more nuanced changes, we aimed to evaluate a fundamental, clinically familiar outcome measure within a realistic, hospital-based timeframe.

### 2.8. Sample Size

The sample size was determined based on previous pilot studies and a statistical analysis aimed at ensuring sufficient test power. A significance level of α = 0.05 and an expected statistical power of 80% required a minimum of 150 participants. Considering potential dropout, a slightly larger number of patients was recruited. Ultimately, a group of 159 patients meeting the criteria for participation in two control studies qualified for the trial.

### 2.9. Statistical Analysis

All the statistical analyses were conducted using Python 3.12 in the Jupyter Notebook environment, utilizing the pandas library for data management, statsmodels for linear regression, and seaborn and matplotlib for the visualization of the results. Prior to the regression analysis, preliminary descriptive statistics were performed for the quantitative variables, including the mean, median, minimum and maximum values, standard deviation (SD), and quartiles Q1 and Q3. To verify the normality of the distribution of quantitative variables, the Shapiro–Wilk test was applied using the shapiro() function (scipy.stats). To assess the influence of multiple independent variables on dependent variables, multivariate regression using the ordinary least squares (OLS) method was applied. Multivariate regression was conducted separately for two dependent variables: delta Barthel (describing the changes in patient function) and delta Rankin (describing the changes in patient disability levels). The regression models included quantitative variables, such as the BMI, the time since the stroke, and the NIHSS scores, as well as categorical variables, such as the gender, hypertension, diabetes, smoking, and medication use (antihypertensives, antidiabetics, statins, and anticoagulants). The OLS function from the statsmodels library was used to build the models, and standard procedures of this method were applied to interpret the regression coefficients and *p*-values.

## 3. Results

**Figure 1:**
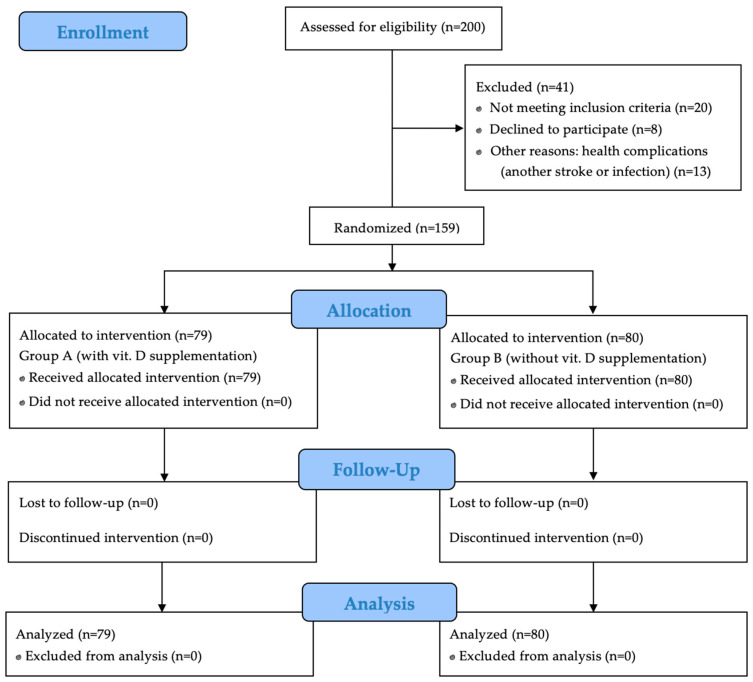
CONSORT 2010 flow chart.

Out of 200 initially enrolled participants, 41 were excluded due to failure to meet the inclusion criteria or refusal to participate. Ultimately, 159 patients qualified for the study. Among them, 51 (32.1%) were women and 108 (67.9%) were men. The participants were randomly assigned to two groups: Group A (vitamin D supplementation, *n* = 79) and Group B (placebo, *n* = 80). Figure 1 presents a detailed study flow chart by CONSORT 2010.

For 42 days, the participants underwent physical therapy using the PNF method and mirror therapy. Blood samples were collected at six-week intervals to measure routine and biochemical parameters. All the tests and surveys were administered by the same clinician.

Table 1 presents the clinical characteristics of the patients, comparing quantitative variables between groups with and without vitamin D supplementation. Statistically significant differences were observed between the groups. The mean BMI was significantly lower in the supplementation group (*p* = 0.046). The post-intervention vitamin D levels were significantly higher in the supplementation group (*p* = 0.004). The change in vitamin D levels (delta) between the groups was highly significant (*p* < 0.001), indicating effective supplementation. Other variables, such as the NIHSS scores or IGF levels, did not differ significantly between the groups. The effect of vitamin D supplementation on changes in the vitamin D and IGF-1 levels, while controlling for baseline values, was investigated. The results indicated that vitamin D supplementation had a significant positive effect on the change in vitamin D levels (β = 3.20, *p* < 0.001), suggesting that supplementation effectively increased vitamin D concentrations over time. Additionally, the baseline vitamin D level was negatively associated with its change (β = −0.124, *p* < 0.001), meaning that individuals with higher initial levels experienced smaller increases. In contrast, vitamin D supplementation had no significant effect on IGF-1 level changes (β = 0.57, *p* = 0.805), implying that IGF-1 dynamics were likely influenced by other factors beyond vitamin D intake.

Table 2 displays comparative analyses of the qualitative variables. Hypertension was significantly more prevalent in the placebo group (67.5%) than in the supplementation group (46.84%) (*p* = 0.013). Similarly, antihypertensive drug use was more frequent in the placebo group (67.5% vs. 45.57%, *p* = 0.009). Smoking was marginally higher in the placebo group (43.75% vs. 27.85%, *p* = 0.054). No significant differences were found regarding diabetes, antidiabetic drugs, or anticoagulant use between groups. However, statin use was significantly higher in the placebo group (*p* = 0.029), particularly among patients over 60.

Table 3 presents the multivariate linear regression results for the Barthel and Rankin scale outcomes. The analysis revealed significant associations between various factors and the functional outcomes post-intervention. Higher Barthel scores were correlated with a better functional status. Conversely, higher Rankin scores indicated a greater disability.

The multivariate regression analysis demonstrated that the BMI, the NIHSS, hypertension, antihypertensive medication, statin use, and anticoagulants negatively impacted the post-stroke functional outcomes, while increased vitamin D levels positively influenced the recovery (*p* < 0.001).

In the analysis of the Barthel scale results after treatment, a higher BMI, higher NIHSS scores, hypertension, and the use of antihypertensive drugs, statins, and anticoagulants had a significant negative impact on the functional status of stroke patients, leading to poorer outcomes. Conversely, an increase in vitamin D levels showed a positive effect, suggesting that higher vitamin D levels were associated with better functional improvement in patients.

In the analysis of the mRS results after treatment, the only significant factor contributing to a greater disability was the use of anticoagulants, indicating a poorer condition in patients taking these medications. Other variables, such as the BMI, the NIHSS, the delta IGF, the delta vitamin D, the gender, hypertension, diabetes, and the use of other medications, did not significantly affect the level of disability after treatment. 

## 4. Discussion

Recent literature suggests the important role of vitamin D3 in various outcomes after a stroke; however, it remains unknown how vitamin D supplementation affects rehabilitation, and it should be noted that it is not only the presence of a deficiency that may impact patients’ functional outcomes. However, it is essential to acknowledge the conflicting evidence regarding its efficacy. A multi-center randomized trial by Momosaki et al. [26] found no significant differences in Barthel index gains or other functional measures between patients receiving vitamin D3 and those given a placebo. These results suggest that vitamin D supplementation alone may not directly enhance rehabilitation outcomes. 

Therefore, in our project, we examined the relationship between the Barthel index and mRS results and the vitamin D3 (25-OH) levels at two time points following an ischemic stroke during the regenerative–compensatory phase. In our study, which ultimately included 159 ischemic stroke patients undergoing rehabilitation during the regenerative–compensatory phase, there were 51 women (32.1%) and 108 men (67.9%), which aligns with epidemiological studies showing that strokes are more commonly observed in men than women [3].

The functional outcomes after six weeks of rehabilitation were negatively affected by the patients’ BMI and NIHSS scores, hypertension, and the use of antihypertensive drugs and statins. The BMI (kg/m^2^) had a significant negative impact on the Barthel score (β = −0.06, *p* = 0.033). This indicates that a higher BMI was associated with poorer functional outcomes after treatment. The higher the patient’s body mass, the smaller the functional improvement. The NIHSS (points), reflecting the severity of neurological symptoms, also showed a significant negative impact on the Barthel score (β = −0.18, *p* = 0.036). Higher NIHSS scores, indicating more severe neurological deficits, were associated with poorer functional outcomes after treatment. An increase in vitamin D levels had a significant positive impact on the Barthel score (β = 0.07, *p* = 0.006). This suggests that a greater increase in vitamin D levels was associated with a better functional status after treatment, indicating that vitamin D may have a beneficial effect on stroke recovery. Despite the relatively short duration, our patients demonstrated measurable changes in their Barthel index scores, suggesting that, even within six weeks, improvements in ADLs are detectible—particularly among those receiving vitamin D supplementation. Nonetheless, we acknowledge that longer follow-ups might reveal more robust functional gains.

A recent randomized controlled trial by Gupta et al. [27] demonstrated the potential benefits of vitamin D and calcium supplementation in ischemic stroke survivors with a vitamin D deficiency or insufficiency. Their study revealed that patients receiving vitamin D and calcium were more likely to achieve favorable outcomes on the mRS at six months compared to those receiving the usual care alone. Notably, the survival probability was significantly higher in the intervention group (83.8%) compared to the control group (59.5%) (*p* = 0.049), suggesting that vitamin D supplementation may contribute to improved long-term functional outcomes and a reduced mortality in post-stroke patients. These findings align with our results, emphasizing the role of vitamin D in enhancing recovery during the regenerative–compensatory phase. Although our study focused on shorter-term outcomes (six weeks), the evidence from Gupta et al. highlights the need for extended follow-up periods to assess the durability of these benefits. This reinforces the importance of addressing vitamin D deficiencies as part of comprehensive post-stroke care. Future trials with larger cohorts and longer follow-ups are warranted to further substantiate these findings and determine the optimal supplementation protocols for stroke rehabilitation.

Borowicz et al. [28] also demonstrated in their study that a patient’s age influenced the functional outcomes assessed using the Barthel scale. An older age (B = −0.01, *p* = 0.04) was associated with lower daily activity, whereas higher vitamin D levels improved daily activity and the Barthel index scores (B = −0.02, *p* = 0.01). A single-factor linear regression analysis assessed the effect of selected variables on daily activities (Barthel scale). The influence of age showed that an older age (B = −0.01, *p* = 0.04) was associated with poorer daily activity, whereas higher vitamin D levels improved this activity (B = −0.02, *p* = 0.01).

Similarly, Hesami et al. [13] conducted a prospective, double-blind, randomized clinical trial evaluating the effect of a single intramuscular dose of 600,000 IU of vitamin D3 in ischemic stroke patients undergoing rehabilitation. The functional outcomes were assessed using the Barthel scale, similar to our project. The study group ultimately included 40 patients, and the final evaluation was conducted after three months. This study concluded that a single intramuscular dose of 600,000 IU of vitamin D3 might have a neuroprotective effect in patients with a moderate ischemic stroke, showing significant improvement in the NIHSS and Barthel scale outcomes.

Similarly to our project, Sari et al. [29] evaluated the effect of vitamin D treatment on rehabilitation in patients with hemiparesis in a randomized, double-blind study. The study involved 63 ischemic stroke patients divided into two groups. Group A (31 patients) received 300,000 IU of intramuscular vitamin D before rehabilitation, while group B (32 patients) received saline. Each patient underwent assessments using the Barthel and Berg scales at the start and three months later. The authors found a significant improvement in the daily activities of patients receiving vitamin D3, although there was no significant improvement in mobility.

This study confirmed that vitamin D supplementation had a positive effect on improving the balance and daily activities in patients with hemiparesis after an ischemic stroke who had low vitamin D levels. However, a direct comparison with our study is challenging due to differences in the timing of rehabilitation, group size, and method of vitamin D administration. In our project, all the patients underwent rehabilitation during the regenerative–compensatory phase, which is the most favorable period for brain plasticity and recovery. Our study group received a daily oral supplementation of 2000 IU of vitamin D for four weeks, unlike the single dose in the study by Sari et al. [29].

A multivariate linear regression analysis of the Barthel scale results post-treatment revealed significant relationships between various variables and the patients’ functional status after the intervention. In our analysis, a higher BMI, higher NIHSS scores, hypertension, and the use of antihypertensive drugs, statins, and anticoagulants had a significant negative impact on the post-stroke functional status, leading to poorer outcomes. In contrast, an increase in vitamin D levels (delta_d3) showed a positive effect, suggesting that higher vitamin D levels are associated with better functional improvement.

Utkan-Karasu et al. [30] also evaluated the effect of vitamin D supplementation on the lower-limb motor function in stroke patients. Their study included 76 patients hospitalized for stroke rehabilitation between May 2018 and February 2020. The patients were divided into two groups – those who received vitamin D supplementation and those who did not. Their motor function and mobility were compared using the Brunnstrom recovery stages and the functional ambulation classification (FAC) before and after rehabilitation. One group of 39 patients received vitamin D during rehabilitation, and 37 did not. Both groups were similar in age, gender, stroke type, comorbidities, and FAC/Brunnstrom scores at baseline (*p* > 0.05). By the end of rehabilitation, changes in the FAC and Brunnstrom scores were greater in the patients receiving vitamin D supplementation (*p* = 0.005 and *p* = 0.018). However, vitamin D did not affect the patients rehabilitated outside the regenerative–compensatory period. This research closely aligns with our project regarding oral vitamin D supplementation. Stroke patients in both studies experienced improved mobility and motor function, indicating the importance of vitamin D in enhancing the post-stroke rehabilitation outcomes.

The findings of this study align with the growing body of evidence highlighting the positive effects of vitamin D supplementation on the functional outcomes in stroke rehabilitation. A systematic review by Fleet et al. demonstrated that vitamin D supplementation was associated with improvements in motor function, mobility, and stroke impairment across various randomized controlled trials and non-randomized interventions. However, despite these promising results, the review underscored the lack of consistent evidence supporting improvements in the activities of daily living. The variability in dosing regimens, patient populations, and outcome measures across studies limits the generalizability of these findings. This reinforces the importance of addressing vitamin D deficiencies as part of post-stroke care while emphasizing the need for further large-scale, methodologically robust trials to elucidate the optimal supplementation protocols and their long-term benefits. Future research should aim to standardize treatment protocols and explore the potential synergistic effects of vitamin D supplementation with comprehensive rehabilitation programs to maximize the recovery outcomes [31].

### Study Limitations

This study has several limitations. First, it was conducted at a single center, which may limit the generalizability of the results. Multi-center trials are necessary to confirm the findings. Additionally, this study evaluated only one dose of vitamin D3, and different dosages or administration durations may yield varying outcomes. We did not account for external factors such as sunlight exposure, dietary intake, or seasonal variations that could affect vitamin D levels. The observation period was limited to six weeks, which may not have captured the long-term effects of vitamin D supplementation on the functional outcomes. Larger sample sizes and extended follow-up periods are recommended for future studies.

## 5. Conclusions

Vitamin D3 supplementation positively influenced the functional outcomes in patients undergoing rehabilitation after an ischemic stroke. Higher increases in vitamin D levels were associated with better improvements in Barthel index scores, suggesting a potential role of vitamin D in enhancing neuroplasticity and recovery during the regenerative–compensatory phase. While the results are promising, further large-scale, multi-center studies are required to fully understand the long-term impact and to optimize vitamin D supplementation strategies in stroke rehabilitation.

## Figures and Tables

**Table 1:** Clinical characteristics of patients—comparison of quantitative variables between groups with and without vitamin D supplementation.

Variable	Group A				Group B				*p*-Value	*p*-Value *
	Mean	Min	Max	SD	Mean	Min	Max	SD		-
Weight (kg)	77.48	52	99	10.62	74.11	46	108	14.92	0.08	-
BMI (kg/m^2^)	24.84	17.24	31.14	2.49	26.01	16.33	35.44	4.32	0.05	-
NIHSS (points)	16.1	10	18	1.12	16.3	14	19	1.31	0.53	-
IGF—baseline (ng/mL)	120.93	49.9	238	48.1	122.67	49.9	238	45.39	0.69	-
IGF—follow-up (ng/mL)	137.37	64.9	247	48.27	138.49	61.1	247	47.19	0.79	-
Vitamin D baseline	28.14	18	60.1	9.35	26.78	7.9	64.3	11.91	0.17	-
Vitamin D follow-up	30.67	19.9	62.2	9.25	26.28	8	56.2	10.87	0.004	-
Delta IGF (ng/mL)	16.43	−24	80	13.44	15.82	−19	80	15.42	0.51	0.57 (*p* = 0.805)
Delta vitamin D	2.53	0.2	11.6	1.71	−0.5	−24.3	15.7	5.34	<0.001	3.20 (*p* < 0.001)

Notes: Group A, with vit. D supplementation; Group B, without vit. D supplementation. * Regression β.

**Table 2:** Demographic and clinical characteristics of patients—comparison of qualitative variables between groups with and without vitamin D supplementation.

Variable	Category	Group A		Group B		*p*-Value
Gender	Female	26	32.91%	25	31.25%	0.957
	Male	53	67.09%	55	68.75%	
Hypertension	No	42	53.16%	26	32.5%	0.013
	Yes	37	46.84%	54	67.5%	
Diabetes	No	60	75.95%	55	68.75%	0.402
	Yes	19	24.05%	25	31.25%	
Smoking	No	57	72.15%	45	56.25%	0.054
	Yes	22	27.85%	35	43.75%	
Antihypertensive drugs	No	43	54.43%	26	32.5%	0.009
	Yes	36	45.57%	54	67.5%	
Antidiabetic drugs	No	59	74.68%	56	70%	0.629
	Yes	20	25.32%	24	30%	
Statins	No	1	1.27%	6	7.5%	0.029
	Yes	78	98.73%	74	92.5%	
Anticoagulants	No	3	3.8%	6	7.5%	0.505
	Yes	76	96.2%	74	92.5%	

Notes: Group A, with vit. D supplementation; Group B, without vit. D supplementation.

**Table 3:** Multivariate linear regression results for the dependent variables delta Barthel and delta Rankin, including various clinical and demographic factors.

Independent Variable	Coefficient	Std Err	t-Value	*p*-Value	CI Lower	CI Upper
Results of multivariate linear regression for the dependent variable delta Barthel						
NIHSS (points)	−0.09	0.09	−0.99	0.324	−0.27	0.09
Delta vitamin D (ng/mL)	−0.10	0.03	−3.90	<0.001	−0.15	−0.05
Hypertension (yes/no)	0.74	0.22	3.40	<0.001	0.31	1.16
Smoking (yes/no)	0.42	0.23	1.85	0.066	−0.03	0.88
Antidiabetic drugs (yes/no)	0.52	0.24	2.11	0.037	0.03	1.00
Anticoagulants (yes/no)	0.04	0.02	1.63	0.104	−0.01	0.09
BMI (kg/m^2^)	−0.02	0.01	−1.54	0.127	−0.04	0.00
Delta IGF (ng/mL)	0.00	0.01	−0.08	0.933	−0.02	0.01
Gender (0 = female, 1 = male)	−0.04	0.08	−0.42	0.672	−0.20	0.13
Diabetes (yes/no)	−0.03	0.09	−0.35	0.724	−0.20	0.14
Antihypertensive drugs (yes/no)	−0.06	0.08	−0.71	0.477	−0.21	0.10
Statins (yes/no)	−0.02	0.01	−1.81	0.072	−0.03	0.00
Results of multivariate linear regression for the dependent variable delta Rankin						
BMI (kg/m^2^)	−0.02	0.01	−1.54	0.127	−0.04	0.00
NIHSS (points)	−0.06	0.03	−1.83	0.069	−0.12	0.00
Delta IGF (ng/mL)	0.00	0.00	1.16	0.248	0.00	0.01
Delta vitamin D (ng/mL)	0.04	0.01	4.28	<0.001	0.02	0.05
Gender (0 = female, 1 = male)	−0.04	0.08	−0.42	0.672	−0.20	0.13
Hypertension (yes/no)	−0.06	0.08	−0.75	0.453	−0.21	0.10
Diabetes (yes/no)	−0.03	0.09	−0.35	0.724	−0.20	0.14
Smoking (yes/no)	−0.09	0.08	−1.17	0.245	−0.25	0.07
Antihypertensive drugs (yes/no)	−0.06	0.08	−0.71	0.477	−0.21	0.10
Antidiabetic drugs (yes/no)	−0.03	0.09	−0.35	0.724	−0.20	0.14
Statins (yes/no)	−0.02	0.01	−1.81	0.072	−0.03	0.00
Anticoagulants (yes/no)	−0.01	0.01	−0.71	0.477	−0.02	0.01

Abbreviations: CI, confidence interval; NIHSS, National Institutes of Health Stroke Scale; IGF, insulin-like growth factor.

## Data Availability

The raw data supporting the conclusions of this article will be made available by the authors upon request.

## Abbreviations

The following abbreviations are used in this manuscript:

25(OH)D	25-hydroxyvitamin D
BDNF	brain-derived neurotrophic factor
BI	Barthel index
BMI	body mass index
CI	confidence interval
CONSORT	Consolidated Standards of Reporting Trials
CT	computed tomography
DoH	Declaration of Helsinki
ECG	electrocardiography
FAC	functional ambulation classification
GCP	good clinical practice
IGF	insulin-like growth factor
IL-6	interleukin 6
mRS	modified Rankin scale
MRI	magnetic resonance imaging
NDT	neuro-developmental treatment
NIHSS	National Institutes of Health Stroke Scale
NGF	nerve growth factor
OLS	ordinary least squares
PNF	proprioceptive neuromuscular facilitation
RCT	randomized controlled trial
SD	standard deviation
TNF-α	tumor necrosis factor alpha
VDR	vitamin D receptors
